# Pulmonary Angiosarcoma With Synchronous Invasive Aspergillosis Presenting as Diffuse Alveolar Hemorrhage and Acute Kidney Injury: A Case Report of a Previously Unreported Combination Posing a Diagnostic Challenge

**DOI:** 10.7759/cureus.38507

**Published:** 2023-05-03

**Authors:** Prabasha Weeraddana, Manbir K Sandhu, Sohini Anand, Hedaya Othman, Mina Makar, Bhavna Matta

**Affiliations:** 1 Internal Medicine, Danbury Hospital, Danbury, USA; 2 Pathology, Danbury Hospital, Danbury, USA

**Keywords:** hemoptysis, acute kidney failure, ground-glass opacities, alveolar hemorrhage, hypoxic respiratory failure, invasive pulmonary aspergillosis, pulmonary angiosarcoma

## Abstract

Angiosarcoma is a rare soft tissue sarcoma originating from endothelial cells. It can occur anywhere when there is a blood vessel or lymphatic channel, making highly perfused cutaneous sites their usual location, though they can also develop within visceral structures. Pulmonary angiosarcoma is usually caused by metastasis from other primary sites. The clinical course of pulmonary angiosarcoma is very aggressive, and the prognosis is poor. We present a case of a 55-year-old man who presented to the hospital with progressive exertional dyspnea and right-sided pleuritic chest pain for the past few days. He was found to have recurrent anemia and acute kidney injury. His hospital course was complicated by the development of hypoxia and hemoptysis. Computed tomography of the chest without contrast revealed bilateral nodular, ground-glass opacities compatible with diffuse alveolar hemorrhage. Further investigation with a lung biopsy revealed epithelioid angiosarcoma with extensive microvascular tumor emboli and invasive pulmonary aspergillosis (*Aspergillus fumigatus*) with patchy necrotizing pneumonia. He later developed acute hypoxic respiratory failure and worsening kidney failure, so he was transferred to the intensive care unit. Upon discussing with the family, the patient was put on comfort measures, and he passed away the following day. We present a rare presentation of concurrence of pulmonary angiosarcoma and invasive aspergillosis. Upon searching the literature, our case is one of the first to report such concurrence. Because of its rarity, the non-specific clinical presentation makes the diagnosis challenging.

## Introduction

Angiosarcoma is a rare cancer originating from endothelial cells lining the blood or lymphatic vessels and can arise anywhere in the body. Pulmonary angiosarcoma includes both primary and metastatic tumors. The occurrence rate of pulmonary angiosarcoma ranges from 0.001% to 0.030% [1]. It mainly affects middle-aged males. The period of survival after surgery is only 12 months [2]. Invasive pulmonary aspergillosis is primarily seen in immunocompromised patients. Epithelioid angiosarcoma of the lung co-existing with invasive aspergillosis is a rare combination that has never been reported in the existing literature. Because of its rarity, the non-specific clinical presentation makes the diagnosis challenging. Here, we describe a case of a 55-year-old immunocompromised male who presented with progressive exertional dyspnea and right-sided pleuritic chest pain for the past few days. Later, he was found to have epithelioid angiosarcoma with extensive microvascular tumor emboli and invasive pulmonary aspergillosis (*Aspergillus fumigatus*) with patchy necrotizing pneumonia. Unfortunately, he rapidly deteriorated with the development of acute hypoxic respiratory failure and worsening kidney failure. The patient passed away after transitioning to comfort care.

## Case presentation

A 55-year-old Caucasian male presented to the emergency department (ED) complaining of right-sided pleuritic chest pain and progressive exertional dyspnea for the past few days. He has had multiple hospital admissions for anemia in the last two months, requiring several transfusions and extensive workups. Table 1 summarizes immune and infectious workups, which were all negative. The workup included a bone marrow biopsy negative for the lymphoproliferative disorder. The patient was a non-smoker. He drank alcohol occasionally and denied asbestos exposure or intravenous (IV) drug use. He had a past medical history of systemic lupus erythematosus (SLE) with lupus nephritis, leading to end-stage renal disease and a kidney transplant twice (2004 and 2016). The patient also had a history of polymyositis, heart failure, coronary artery disease, and hypertension, among other comorbidities. The patient was on tacrolimus 0.4 mg daily, mycophenolate mofetil (MMF) 720 mg twice daily, and prednisone 5 mg daily.

**Table 1:** Immune and infectious workups done during past hospitalizations. PCR: polymerase chain reaction.

Test name	Results
Antinuclear antibodies (ANA)	1.:40 (<1:40)
Direct antiglobulin test (DAT)	Negative
Anti-double-stranded DNA antibody	<1 ​​IU/mL (0-4 IU/mL)
Paroxysmal nocturnal hemoglobinuria (PNH) immunophenotyping	Normal immunophenotyping results. No PNH clone is detected in RBC, granulocytes, or monocytes
Anti-tissue transglutaminase antibodies	<0.8 U/mL (0-15 U/mL)
Beta-D glucan	<31 pg/mL (0-60 pg/mL)
Hemoglobin electrophoresis	No electrophoretic evidence of abnormal hemoglobin or beta-thalassemia
Direct Coombs test	Negative
Parvovirus serology	Not detected
Cytomegalovirus PCR	Not detected
Anaplasma PCR	Not detected
Babesia PCR	Not detected

On presentation to the ED, the patient was afebrile with a heart rate between 100 and 110 beats per minute (BPM), a respiratory rate between 20 and 24 breaths per minute, and an oxygen saturation of 98% on room air. Upon physical examination, he appeared pale. The lungs were clear, with no wheezes, rales, or rhonchi. Bilateral lower extremity edema was present, which was pitting up to the mid-thigh level. Laboratory work-up showed a white blood cell (WBC) count of 6.9 × 109/L, a hemoglobin concentration of 6.1 g/dl, a platelet count of 181 × 109/L, and serum creatinine (Scr) levels of 2.36 mg/dl (baseline Scr = 1.5-1.6 mg/dl). Given the examination and lab findings, the patient was admitted for further investigation of recurrent anemia and acute kidney injury (AKI).

While in the hospital, he received 11 units of packed red blood cells (RBCs) over 22 days for recurrent anemia. A gastroenterologist was consulted, and the patient underwent an endoscopy and colonoscopy, which were both normal. Furthermore, the biopsies were negative for malignancy. His anti-centromere antibody (Ab), JO-1 antibody, ribonucleic protein antibody, anti-Scl-70 Ab, anti-Smith Ab, anti-Sjogren's syndrome A (anti-SS-A) antibody, anti-Sjogren's syndrome B (anti-SS-B) antibody, myeloperoxidase antibody, and proteinase 3 antibody tests came back negative. The (1,3)-beta-D-glucan test was also negative for *Aspergillus*.

**Figure 1:**
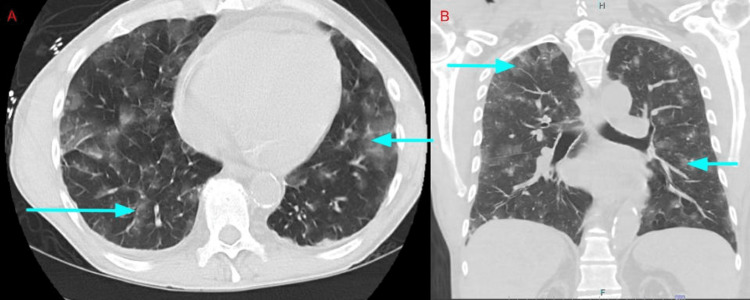
CT of the chest without contrast revealing bilateral nodular ground-glass opacities (blue arrows pointing to ground-glass opacities) compatible with alveolar hemorrhage.

The hospital course was complicated by the development of hemoptysis and hypoxia on the 14th day. He underwent computed tomography (CT) of the chest without contrast, revealing bilateral nodular ground-glass opacities compatible with alveolar hemorrhage (Figure 1).

**Figure 2:**
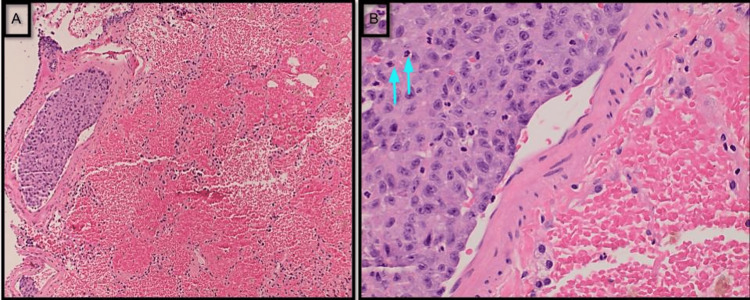
Right lung wedge biopsies showed epithelioid angiosarcoma with microvascular tumor emboli (A: H&E, x40; B: H&E, x2) (blue arrows showed mitotic figures). H&E: Hematoxylin and eosin stain.

**Figure 3:**
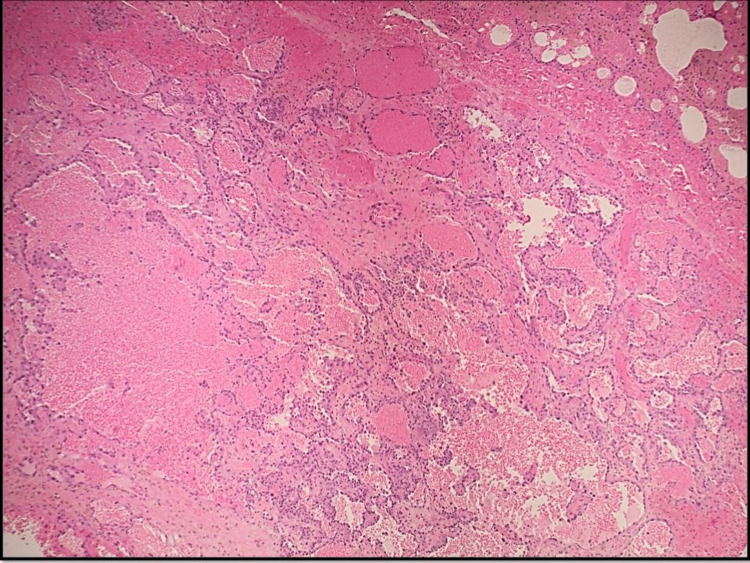
Right lung wedge biopsies showed fresh alveolar hemorrhage and complex vascular structures (hematoxylin and eosin, x2).

**Figure 4:**
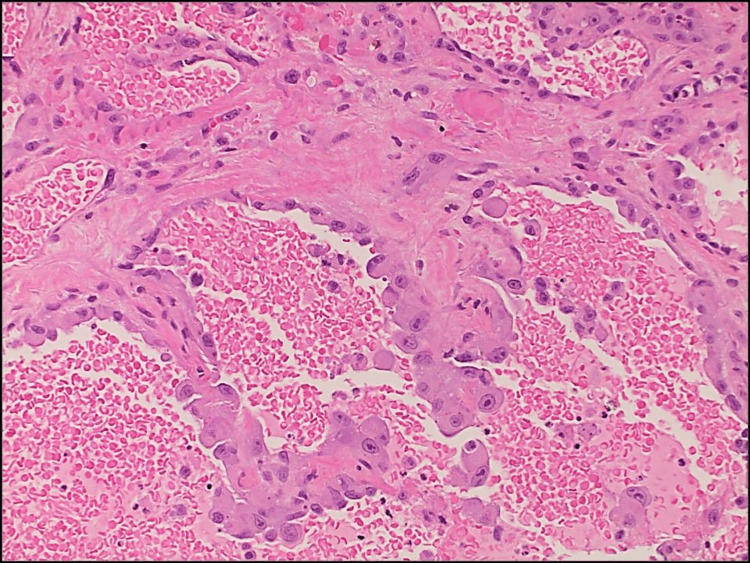
Right lung wedge biopsies depicted epithelioid angiosarcoma showing irregular complex vascular structures lined by malignant epithelioid endothelial cells (hematoxylin and eosin, x40).

**Figure 5:**
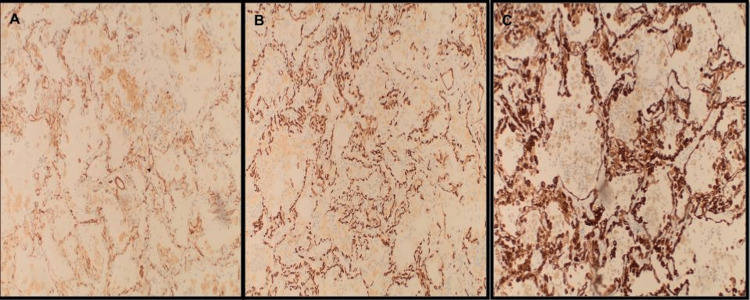
Immunohistochemical staining of lung wedge biopsies demonstrated strong immunoreactivity of the tumor cells for CD31 (A) and cytokeratin CAM 5.2 (C) and patchy cytoplasmic staining for CD34 (B).

**Figure 6:**
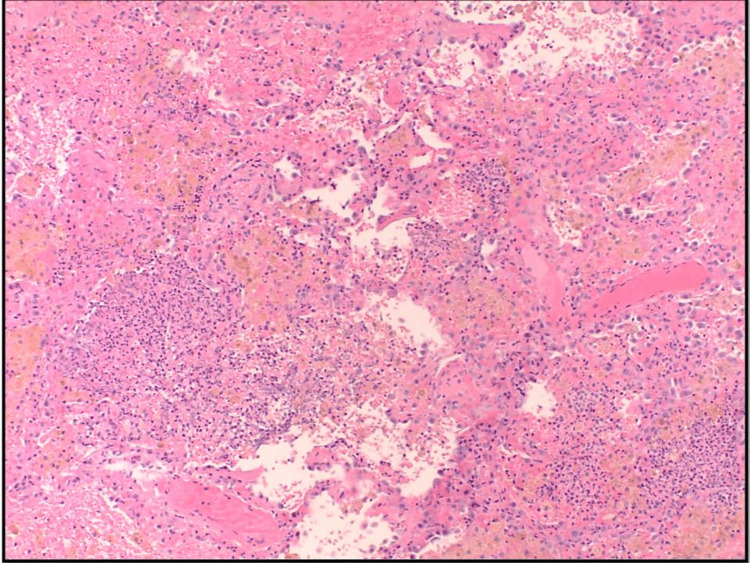
Right lung wedge biopsies revealed necrotizing pneumonia with neutrophilic microabscesses (hematoxylin and eosin, x20).

**Figure 7:**
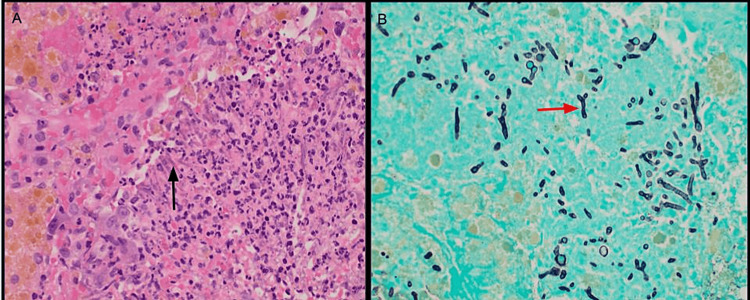
(A) Hematoxylin and eosin (H&E) stain of lung wedge biopsies showing fungal hyphae (black arrow) within a focus of necrotizing bronchopneumonia. (B) Grocott’s methenamine silver stain (GMS) of lung wedge biopsies showing septate hyphae with 45-degree angle branching (red arrow), morphologically consistent with Aspergillus species.

The pulmonology department was consulted, and he was started on broad-spectrum antibiotics with IV ceftriaxone and steroids with Solu-Medrol 60 mg every six hours. This was a challenging situation because more aggressive immunosuppression would be reasonable if this were due to immune-mediated vasculitis. However, more immunosuppression will aggravate the disease if this is due to infection. After further discussion with a rheumatologist and nephrologist, MMF was held, and intravenous immunoglobulins were started (intravenous immunoglobulins do not suppress the immune system, and hence do not make it more difficult to get over infections), which mildly improved the anemia. His course was further complicated by a worsening AKI. Urinary analysis showed proteinuria, hematuria, elevated serum creatinine, and diffused alveolar hemorrhage concerning pulmonary-renal syndrome. The patient underwent video-assisted thoracoscopy surgery (VATS) with right lung wedge biopsies, revealing epithelioid angiosarcoma with extensive microvascular tumor emboli and invasive pulmonary aspergillosis (*Aspergillus fumigatus*) with patchy necrotizing pneumonia, and an extensive fresh and organizing alveolar hemorrhage (Figure 2). Histology of the lung wedge biopsy also showed parenchymal and visceral pleural nodules composed of large epithelioid tumor cells arranged in nests, cords, and complex inter-anastomosing vascular channels and extensive fresh and organizing alveolar hemorrhage with hemosiderin-laden macrophages, but there was no evidence of vasculitis (Figures 3, 4). Immunohistochemical stains demonstrated strong immunoreactivity of the tumor cells for CD31 and cytokeratin CAM 5.2, patchy cytoplasmic staining for CD34, and an absence of staining for cytokeratin 7, cytokeratin 20, thyroid transcription factor 1 (TTF-1), and napsin A (Figure 5). This immunoreactivity pattern was consistent with an epithelioid angiosarcoma. The origin of this angiosarcoma is uncertain. Possible primary sites include the lung, bone, liver, and spleen. Work-up also revealed patchy necrotizing bronchopneumonia with invasive septate hyphae with 45-degree angle branching, morphologically consistent with *Aspergillus* species (Figures 6, 7). This was confirmed by a positive Grocott methenamine silver (GMS) staining of the fungal hyphae and a negative stain for acid-fast bacilli (AFB).

The infectious disease department was consulted, and the patient was started on voriconazole per their recommendation. He shortly developed acute hypoxic respiratory failure requiring a high-flow nasal cannula (HFNC). He was also noted to be in atrial fibrillation with a rapid ventricular rate of 150. He was transferred to the intensive care unit (ICU) and started on an amiodarone infusion at 1 mg/min. Later, he was put on bilevel-positive airway pressure (BiPAP) for increased work of breathing. His AKI continued to worsen with increasing volume overload. He had a poor response to diuretics, so he was started on dialysis; however, dialysis was shortly terminated due to hypotension. Given the patient's chronically immunocompromised state and critical status, he was transitioned to comfort measures only (CMO) after a discussion with the family, and the patient passed away the next day.

## Discussion

Angiosarcoma of the lungs is a rare cancer that makes up 0.001-0.03% of all cancer cases [3,4]. Pulmonary angiosarcoma resulting from metastasis is common [5] and is usually referred to as secondary pulmonary angiosarcoma. Along with the liver and lymph nodes, the lung is one of the most typical sites for metastatic involvement. A total of 60% to 80% of cutaneous and cardiac angiosarcomas metastasize to the lungs. Therefore, a primary site outside the lung should be examined when angiosarcoma in the lung is diagnosed [6]. Albores-Saavedra et al. concluded that an average age of 73 years is the presentation age for angiosarcoma, which primarily affects older adults [7]. It has also been concluded that white people are more likely to develop them [7]. By a ratio of 3:1, men tend to be afflicted with this neoplasm more than women [8]. Respiratory symptoms that patients commonly report are hemoptysis, cough, dyspnea, and pleuritic chest pain, in addition to weight loss. Primary angiosarcoma can appear as solitary or multifocal lesions on CT scans [9,10]. Hemoptysis is the most frequent presenting symptom in pulmonary angiosarcoma, which can occasionally be unrelenting and protracted [11,12].

Our patient initially presented with dyspnea and pleuritic chest pain and was found to have acute-on-chronic anemia. While investigating the cause of anemia, the patient developed hemoptysis with hypoxia. Given this non-specific clinical presentation, investigations led to different paths, complicating the diagnosis workup.

Multiple solid nodular lesions in CT are a common finding in secondary angiosarcoma of the lungs [13]. Shimabukuro et al.'s review of 31 cases of primary pulmonary angiosarcoma included pulmonary nodules (87%), infiltrations (22%), ground-glass opacity (GGO) (13%), pleural effusion (16%), pulmonary nodules surrounded by GGO (9%), and the invasion of other organs (19%) as the most prominent CT features [14]. When our patient started having hemoptysis, he underwent a CT of the chest, which showed bilateral nodular ground-glass opacities. These findings can be seen in diffuse pulmonary hemorrhage, secondary to systemic disorders such as vasculitis in lupus pneumonitis or Goodpasture syndrome, or coagulation disorders and opportunistic infections, especially in the patient's state of immunosuppression secondary to a history of renal transplant and immunosuppressant use. Because the bronchoscopy was negative for all infectious etiologies, our main suspicion was vasculitis secondary to an autoimmune process. He underwent extensive autoimmune and infectious workups, all of which were negative. In the meantime, the patient developed hypoxia requiring oxygen. Given his AKI and CT findings of GGO with alveolar hemorrhage, pulmonary-renal syndrome was suspected. He underwent a lung wedge biopsy, which finally yielded the diagnosis of pulmonary angiosarcoma.

The patient was also diagnosed with invasive pulmonary aspergillosis (IPA). This describes a rare instance of the co-occurrence of invasive aspergillosis and secondary angiosarcoma of unknown primary origin. The illnesses caused by *Aspergillus *species include invasive infections, chronic pulmonary diseases, and allergy syndromes. For patients with compromised immune systems, invasive aspergillosis is a major cause of morbidity and mortality [15,16]. The use of corticosteroids, renal transplantation, and immunosuppressive medications for his kidney transplant may have contributed to the development of invasive aspergillosis in this patient [16]. Interestingly, this patient's (1,3)-beta-D-glucan test, which is widely used as a biomarker for early invasive aspergillosis, was negative. It was diagnosed only after a lung wedge biopsy revealed the characteristic septate hyphae on histology. IPA is still best treated with the oral and intravenous formulations of voriconazole [17]. Radiation, chemotherapy, and surgical resection have all been suggested as possible treatment options for angiosarcoma [9]. Several prior studies have shown that angiosarcoma is radiosensitive [5]. Unfortunately, our patient rapidly deteriorated, developing acute hypoxic respiratory failure before starting treatment for angiosarcoma.

Early diagnosis of pulmonary angiosarcoma is challenging, given overlapping symptoms with other clinical conditions and non-specific radiological findings. A lung biopsy is essential for a definitive diagnosis.

## Conclusions

The combination of invasive aspergillosis and angiosarcoma is an extremely rare occurrence. To the best of our knowledge, there has not been any reported case of pulmonary angiosarcoma with synchronous invasive aspergillosis, even though there are separate cases of each condition in the literature. Our case illustrates the diagnostic challenge this rare clinical anomaly poses. This case will help clinicians be more aware of this condition and have a high suspicion of pulmonary angiosarcoma in patients with similar presentations.
